# High Prevalence and Socioeconomic Inequalities of Mental Disorders Among Adults with Disabilities in Western Iran

**DOI:** 10.1192/j.eurpsy.2025.1495

**Published:** 2025-08-26

**Authors:** B. Piroozi, K. Qaderi Bagajan

**Affiliations:** 1 Social Determinants of Health, Research Center, Research Institute for Health Development, Kurdistan University of Medical Sciences, Sanandaj; 2 Department of Clinical and Health Psychology, Shahid Beheshti University, Tehran, Iran, Islamic Republic Of; 3 Psychological Diagnostics group, Department of Psychology, Humboldt-Universität zu Berlin, Berlin, Germany

## Abstract

**Introduction:**

There is very little information about the mental health status of people with disabilities in Iran, while monitoring the status of this index is very necessary to properly address problems, design appropriate interventions, and evaluate the impact of interventions.

**Objectives:**

in this regard, this study investigated the prevalence of mental disorders among adults with physical and sensory disabilities in Sanandaj City, Iran.

**Methods:**

This descriptive-analytical and cross-sectional study was conducted on people with physical and sensory (sight, hearing and speech) disabilities over 18 years of age in Sanandaj city in 2023. A two-part questionnaire was used in order to collect data in this study. The first part consisted of age, sex, type of disability, basic health insurance status, supplementary health insurance, education, employment status, and economic status (wealth assets). The second part included Goldberg’s 28-item questionnaire (GHQ-28) that was used as a screen tool for mental disorders. Data were analyzed using STATA software version 16.0 (Stata Corp, College Station, TX, USA.)

**Results:**

Finally A total of 607 people (response rate: 99%) participated in this study that 317 were men (52.2%) and 290 were women (47.8%). The prevalence of mental disorders suspicion was 56.7% (344 people) and its prevalence based on the severity of the disorder was 29.7% mild, 16.6% moderate and 10.4% severe. Results indicated that over 56% of participants were suspected of having a mental disorder, with the most common being depression and anxiety. Females, younger participants, the unemployed, those without supplementary health insurance, and those from lower economic classes had significantly higher odds of mental disorder suspicion. An uneven distribution was observed, with a disproportionate concentration among the lower economic status group.

**Conclusions:**

The findings highlight a high prevalence of suspected mental disorders among people with disabilities and inequalities in gender, age, employment, insurance coverage, and socioeconomic status. Addressing mental health needs is crucial through targeted screening, prevention programs, and promoting access to appropriate services for this vulnerable population.

**Keywords:**

Disabled individuals, Disability, Mental health, Psychiatric disorders, Psychology, Health inequality, Iran.

**Disclosure of Interest:**

None Declared

